# Functional biomechanical performance of a novel anatomically shaped polycarbonate urethane total meniscus replacement

**DOI:** 10.1007/s00167-015-3632-6

**Published:** 2015-05-14

**Authors:** A. C. T. Vrancken, F. Eggermont, T. G. van Tienen, G. Hannink, P. Buma, D. Janssen, N. Verdonschot

**Affiliations:** Orthopaedic Research Lab, Radboud Institute for Health Sciences, Radboud University Medical Center, Huispost 547, PO Box 9101, 6500 HB Nijmegen, The Netherlands; Orthopaedic Research Lab, Radboud Institute for Molecular Life Sciences, Radboud University Medical Center, Nijmegen, The Netherlands; Laboratory for Biomechanical Engineering, University of Twente, Enschede, The Netherlands

**Keywords:** Meniscal replacement, Implant, Contact pressure, Kinematics, Knee laxity

## Abstract

**Purpose:**

To evaluate the functional biomechanical performance of a novel anatomically shaped, polycarbonate urethane total meniscus implant.

**Methods:**

Five human cadaveric knees were flexed between 0° and 90° under compressive loads mimicking a squat movement. Anteroposterior (AP) laxity tests were performed in 30° and 90° flexion. Meniscal kinematics and knee laxity were quantified using roentgen stereophotogrammetric analysis. Tibial cartilage contact mechanics were determined in 90° flexion. Measurements were repeated for the native medial meniscus, the implant, after total medial meniscectomy and allograft transplantation.

**Results:**

The implant and allograft displayed increased posterior and medial displacements compared to the native meniscus, yet no differences were found between the implant and allograft. Meniscal condition did not affect rotational laxity. Compared to the native joint, AP laxity for the implant was increased in 30° flexion, but not in 90°. The implant reduced the mean contact pressure compared to meniscectomy but could not restore contact pressures to native meniscus levels. Compared to the native meniscus, the implant significantly increased the peak pressure, while the contact area was reduced. Contact mechanics of the implant and allograft were never statistically different.

**Conclusions:**

Biomechanical performance was similar for the implant and allograft. However, both meniscal replacements could not restore outcomes to native meniscus levels or sufficiently improve outcomes after meniscectomy. This was presumably caused by the mobility allowed by the suture-only horn fixation. The similarity of implant and allograft performance suggests that the novel implant has the biomechanical potential to serve as an alternative to meniscal allograft transplantation.

## Introduction

Being involved in 15 % of the knee injuries in an active population, the menisci are amongst the most vulnerable tissues in the knee joint [19]. In the USA, this results in 650,000 meniscus-related surgeries performed annually, most of which encompass (partial) meniscectomy [3]. However, in the long term, 50 % of the meniscectomized patients develop symptomatic osteoarthritis [12]. Currently, meniscal allograft transplantation is the only treatment option for symptomatic total meniscectomy patients. Although allograft transplantation generally relieves pain and improves knee function [11, 18, 23], several studies suggest structural remodelling and shrinkage of the graft tissue, which may compromise its function in the long term [14, 16, 22]. Also, the availability of allografts is restricted by a limited supply and size-matching requirements.

**Fig. 1:**
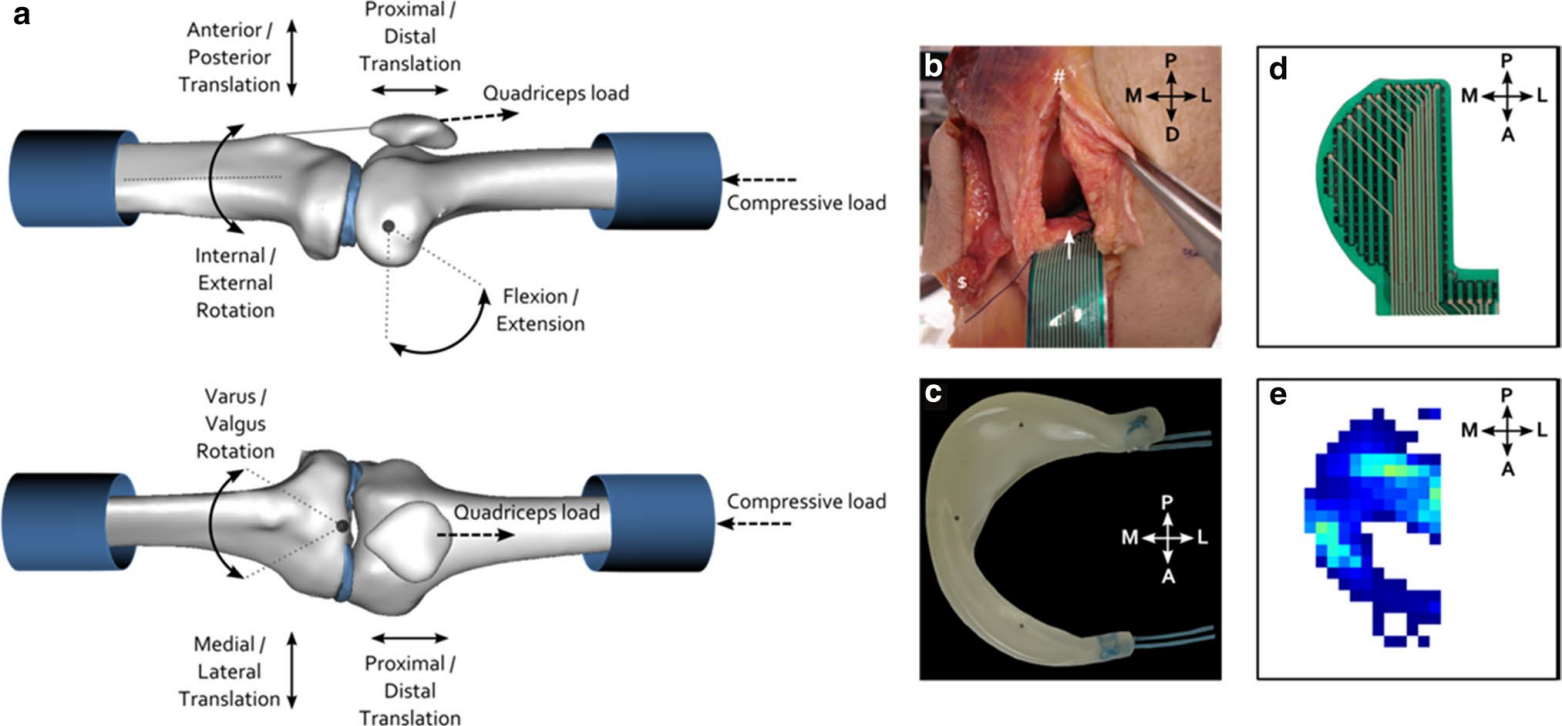
**a** Degrees of freedom and modes of loading allowed by the knee loading rig. The tibia was free to set its varus/valgus rotation, endo/exo rotation, anterior/posterior translation and its medial/lateral translation, while the femur was free to move in proximal/distal translation. Flexion/extension was controlled on the femoral side and fixed during a measurement. An axial compressive load was applied on the femoral side, but always approximately parallel to the tibial longitudinal axis. Extra compression was applied through loading of the quadriceps tendon. **b** Interventions to access the medial knee compartment: a medial parapatellar incision (#) and release of the MCL by isolation of a femoral bone cap ($). Later, the pressure sensor was inserted in between the tibia and the meniscus/meniscal replacements via a 2-cm incision in the anterior meniscotibial ligament (*white arrow*). **c** The PCU implant including the sutures that were used to fix the horns to the tibia plateau. The *black dots* inside the implant are the tantalum beads used to determine meniscal kinematics and knee laxity by roentgen stereophotogrammetric analysis. **d** Tekscan 4010 N pressure sensor. **e** Example of the pressure sensor output. *Dark blue colours* represent low pressures, whereas *light blue* and *green colours* indicate regions with higher pressures

A synthetic, non-resorbable total meniscus replacement could overcome these shortcomings related to the use of meniscal allografts. Since both the fit and fixation of a meniscal replacement have been shown to be important determinants of its functioning [4, 10, 17, 20], we have developed an anatomically shaped, polycarbonate urethane (PCU), total meniscus replacement with extensions for horn fixation to the tibia plateau (Fig. 1b). If this implant is capable of mimicking the native meniscus functions as a load redistributor and a knee stabilizer [5, 20, 27, 32], it may have the potential to put a hold on post-meniscectomy knee pain and functional limitations and ideally also to stop the development of osteoarthritic changes. Therefore, the objective of the current study was to evaluate the functional biomechanical performance of this novel implant and to compare its performance to the clinically relevant control cases. Specifically, whether the implant influenced meniscal kinematics, knee stability and tibial contact mechanics in comparison with the native medial meniscus, after total meniscectomy and meniscal allograft transplantation was assessed.

## Materials and methods

In a knee loading rig, cadaveric knee joints were subjected to a quasi-static loading cycle. Meniscal kinematics and knee stability were evaluated using roentgen stereophotogrammetric analysis (RSA), and tibial contact mechanics were recorded using pressure sensors. The measurements were repeated for the native meniscus, the PCU implant, after total meniscectomy and for a meniscal allograft.

### Implant

Implant geometry was established by segmenting the medial menisci from eight routine knee MR scans of healthy male subjects (aged 21–67) using Mimics (v14.0, Materialise, Leuven, Belgium), and 3D models were created. Meniscal length and width were determined for each 3D model and the model that approached mean dimensions closest was chosen to serve as the implant geometry. The meniscal horns were extended 5 mm to allow for suture fixation to the tibia plateau. Bionate^®^ PCU (grade II 80A, DSM Biomedical, Geleen, Netherlands) implants were subsequently produced through injection moulding. The implants were hydrated in 37 °C saline for 2 weeks prior to testing.

### Specimens

Fresh frozen, left human cadaveric knees were obtained from our institutional Department of Anatomy. Calibrated anteroposterior and lateral knee radiographs were taken to exclude specimens that showed joint space narrowing, osteophytes or chondrocalcinosis. In addition, the images were used to estimate medial meniscus length and width from tibial plateau size measurements [21]. As the prototype implant was available in one size only, knees were excluded from this study when their estimated medial meniscus length or width deviated more than ten per cent from the implant dimensions. In total, ten knees were excluded based on radiographic signs of joint degeneration and seven additional knee joints because of size mismatching. Five male knees (aged 70–88) were included for testing.

### Knee loading rig and specimen preparation

To fit the specimens in the loading rig, the femur and the tibia were transected approximately 15 cm above and below the joint line, while taking care to leave the knee capsule intact. All soft tissues surrounding the knee capsule were left intact, including the full quadriceps tendon. The specimens were then transferred to the knee loading rig, which allowed the joints to move within their natural six degrees of freedom (Fig. 1a) [28]. To align the knee joint, the femur was positioned with its intercondylar notch in the intersection of the flexion/extension axis and the varus/valgus axis, and the tibial shaft was aligned with the internal/external rotation axis of the apparatus. Upon alignment, the specimens were permanently fixed to the holders of the loading rig with polymethyl methacrylate. These holders allowed repositioning of the knee joint without affecting its alignment.

Subsequently, the joint capsule was opened through a medial parapatellar incision. To access the full medial knee compartment, the femoral attachment of the medial collateral ligament (MCL) was released by isolation of a bone cap that could be repositioned with a screw (Fig. 1b). The bone cap technique was chosen to allow easy and reproducible repositioning of the MCL, without changing its kinematics [34]. Three tantalum beads (0.8 mm diameter) were inserted in the periphery of the anterior and posterior horn, and the mid-region of the medial meniscus and the implant (Fig. 1c). One bead (1 mm diameter) was inserted in the tibial origin of the anterior cruciate ligament, to define a reproducible origin of the coordinate system. Four additional tantalum markers (1 mm diameter) were inserted into both the proximal tibia and the distal femur. A metal ring was sutured to the distal end of the rectus femoris tendon, to allow for patellofemoral and tibial load transfer from the loading apparatus.

### Loading protocol

The loading protocol (Table 1) was designed such that we could analyse meniscal displacement and rotational laxity during a simulated squat movement (between 0° and 90° flexion), knee AP laxity in an unloaded knee joint (in 30° and 90° flexion) and cartilage contact pressures under the maximum compressive load (in 90° flexion). Compressive loads in each flexion angle were derived by solving the equilibrium equations belonging to a free body diagram of a squat [31]. Pilot tests demonstrated that the cadaveric tissue could not withstand physiological loads. To prevent tissue damage, the compressive loads, as derived by solving the equilibrium equations, were linearly down scaled to a maximum of 1000 N. For the same reason, loading of the rectus femoris tendon was restricted to 250 N, which was kept constant during all tests. Since the quadriceps loads resulting from the equilibrium equations were larger than 190 N, the quadriceps loads applied in this study should mainly be considered as joint stabilizers.

**Table 1:** Loading protocol describing all loads applied during the testing of one meniscal condition

Flexion angle (°)	Axial compressive load (N)	Quadriceps load (N)	Tibial torque (Nm)	Tibial drawer load (N)
0	357	250	–	–
	357	250	3.4 internal	–
	357	250	3.4 external	–
30	572	250	–	–
	572	250	3.4 internal	–
	572	250	3.4 external	–
	–	–	–	67
60	824	250	–	–
	824	250	3.4 internal	–
	824	250	3.4 external	–
90	1000	250	–	–
	1000	250	3.4 internal	–
	1000	250	3.4 external	–
	–	–	–	67
0	357	250	–	–
	357	250	3.4 internal	–
	357	250	3.4 external	–
90	1000	250	–	–

In each flexion angle, the knee was tested without tibial torque (neutral), and with an internal and external tibial torque of 3.4 Nm [15]. In addition, in 30° and 90° flexion, AP stability of the knee joint was tested through anterior drawer tests. Anterior loads of 67 N were applied to the proximal tibia, to mimic the load that is applied in clinical knee laxity testers [7]. To assess whether repetitive loading of the cadaveric tissue influenced the outcomes of our tests due to tissue degeneration, a second set of measurements in 0° flexion was performed (Table 1).

### Meniscal conditions

**Fig. 2:**
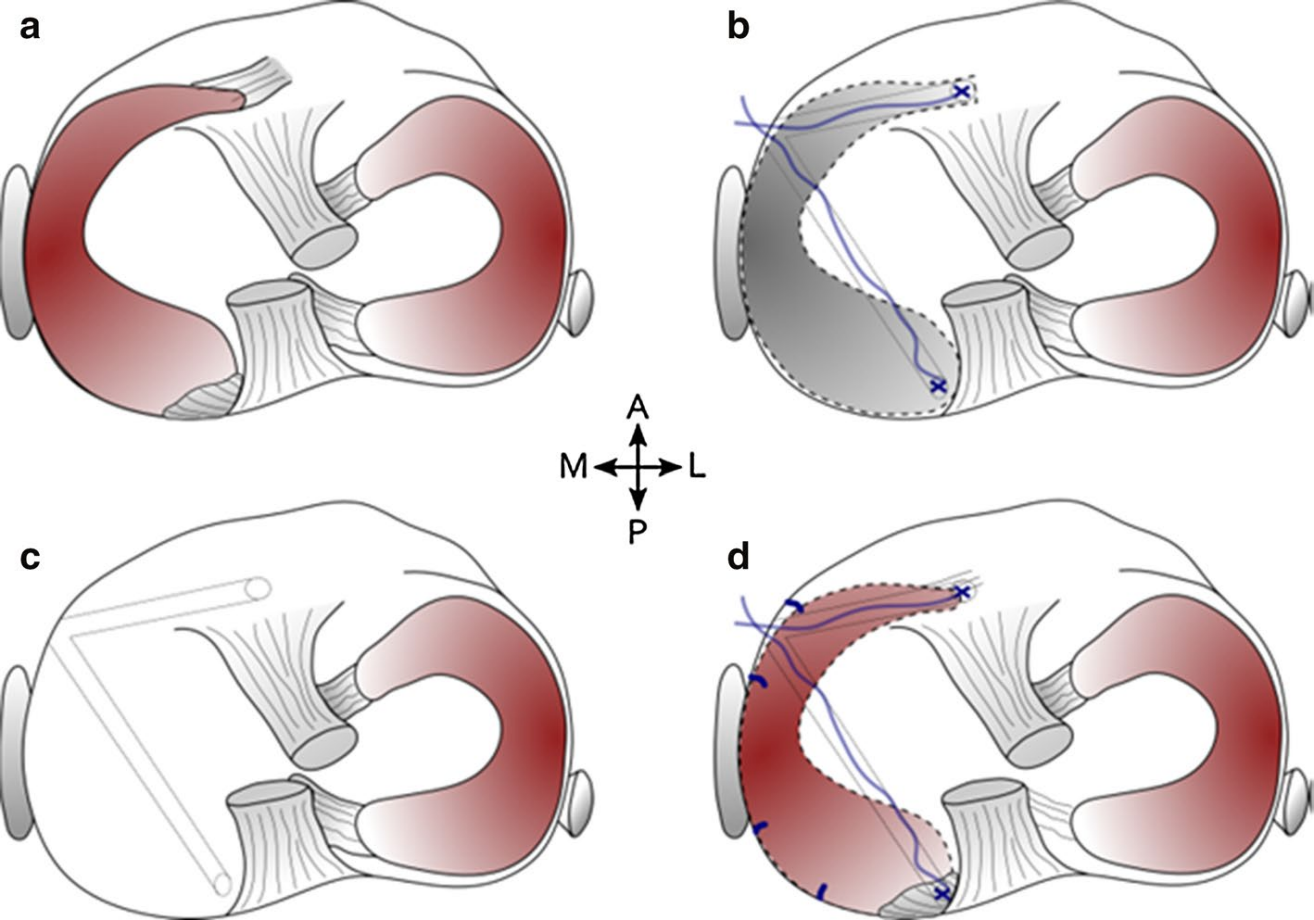
Schematic representation of the tested meniscal conditions. **a** Intact meniscus, **b** PCU meniscal implant, fixed by sutures running through the implant horns and tibial bone tunnels, **c** total meniscectomy and **d** meniscal allograft, fixed by sutures running through the allograft horns and tibial bone tunnels combined with peripheral vertical mattress sutures

Testing of the native meniscus (condition 1, Fig. 2a) was followed by the implant (condition 2, Fig. 2b), a total meniscectomy (condition 3, Fig. 2c) and a meniscal allograft (condition 4, Fig. 2d). To start condition 2, a complete medial meniscectomy was performed. The excised meniscus was wrapped in saline-soaked gauze and frozen at −80 °C until its use as allograft meniscus. Tibial bone tunnels (2.5 mm diameter) were drilled from the anteromedial side of the tibia to the anterior and posterior attachment site of the meniscus on the tibial plateau. Two suture threads (FiberWire #2, Arthrex, Naples, FL, USA) were fixed to each horn of the implant and guided through the tunnels. The implant was positioned and fixed by knotting the sutures together. For testing of condition 3, the implant was removed, and the knee was retested for the meniscectomy condition. Lastly, for condition 4, the tests were repeated for an allograft meniscus, which was obtained from the preceding experiment. As all knees were size matched to the implant, their menisci were regarded to be properly sized allografts as well. The horns of the allograft were fixed in a similar fashion as those of the implant. To comply with the surgical standards that are clinically applied, in addition, the allograft was circumferentially fixed to the joint capsule using four vertical mattress sutures.

### Biomechanical outcome measures

RSA was used to determine meniscal kinematics, knee AP laxity and tibial rotational laxity [26]. For each separate loading step from Table 1, a bi-planar roentgen image was taken. The 3D locations of the tantalum beads inside the tibia, native meniscus, implant and allograft were obtained from each image by relating their 3D positions to the distances known from a calibration image. All tibial beads were superimposed, allowing the determination of the different locations of the meniscus on the tibia for each condition. The meniscal locations were converted to meniscal translations along the AP and ML axes for the flexion intervals 30°–0°, 60°–0° and 90°–0° for the anterior, mid and posterior regions of the meniscus or implant. To determine AP laxity (in 30° and 90° flexion), the centroid of the tantalum beads inside the tibia was determined in unloaded and loaded state. AP laxity was defined as the translation of the centroid along the AP axis. Rotational laxity was defined as the angular rotation of the tibia around its longitudinal axis, as a result of the application of the internal or external torque. The set-up used in our laboratory allowed to determine bead location with an accuracy of 50 μm and repeated measurements can be performed with an accuracy of 200 µm [6].

In 90° flexion, tibial contact mechanics were evaluated using piezo-electric pressure sensors (Fig. 1d, e, K-scan 4010 N, Tekscan, South Boston, MA, USA). To prevent fluid penetration into the sensor, the sensor was placed between two layers of Tegaderm film dressing (3M, St. Paul, MN, USA). After sealing, all sensors were conditioned, equilibrated and calibrated using a materials testing system (MMED, Materials Technology Corporation, La Canada, CA, USA) and a custom calibration tool consisting of two Teflon plates. To optimize the accuracy of the pressure sensor, the calibration curve was derived from a ten point polynomial fit through the calibration points that were equally distributed between 0.9 and 9.0 MPa [8]. Consequently, cartilage contact pressure could be determined with a precision of 0.035 MPa. To allow sensor insertion, the knee was distracted by the weight of the tibia and a 2-cm incision was made to the anterior meniscotibial ligament (Fig. 1b). A suture with a 10″ straight needle (PDS II 2-0, Ethicon, Somerville, NJ, USA) was knotted to the most posteromedial corner of the sensor and guided along the cruciate ligaments. This suture allowed to pull the sensor exactly underneath the meniscus and was re-used to place the sensors for the implant, meniscectomy and allograft conditions in the same position. Additionally, the anteromedial edge of the pressure sensor was fixed by a suture through the knee capsule. A new sensor tab was used for each meniscal condition. Peak pressure, mean pressure and contact area were determined from the pressure maps for each meniscal condition.

### Statistical analysis

A sample size calculation was based on a previous study that evaluated the difference in knee contact mechanics after total medial meniscectomy and subsequent meniscal transplantation [25]. Five knees were required to detect a difference of 1.1 MPa (standard deviation 0.5 MPa) in mean pressure, using a significance level of 0.05 and a power of 80 %.

Linear mixed models were used to study the effect of meniscal condition on contact mechanics, knee laxity and meniscal kinematics. Each outcome measure (peak pressure, mean pressure, contact area, AP laxity in 30° and 90° flexion, internal and external tibial rotation, AP and ML meniscal translation) was analysed separately. The models included specimen as a random factor and all other variables that applied to the outcome measure under analysis (meniscal condition, flexion angle or flexion interval, meniscal region) as fixed factors. A random intercept and slope were included to account for the specimen’s individual response to each experimental condition. For rotational laxity and meniscal displacement, models including the interaction terms between the fixed factors were also evaluated. As the addition of the interaction terms did not significantly increase the model fits based on likelihood ratio tests, the interaction terms were omitted from the models used for the final analysis. Pairwise comparisons between the different levels of the fixed variables were performed by Tukey’s tests that were Bonferroni corrected to account for multiple comparisons. *p* values below 0.05 were considered statistically significant.

## Results

The dimensions of the implant corresponded well to those of the native meniscus. Deviations (mean ± standard deviation) were 4.0 ± 4.1 % for the length and 5.1 ± 4.5 % for the width. Mean size deviations between the allograft and native meniscus were similar; 5.3 ± 4.1 % in length and 4.5 ± 4.6 % in width.

### Meniscal kinematics

**Fig. 3:**
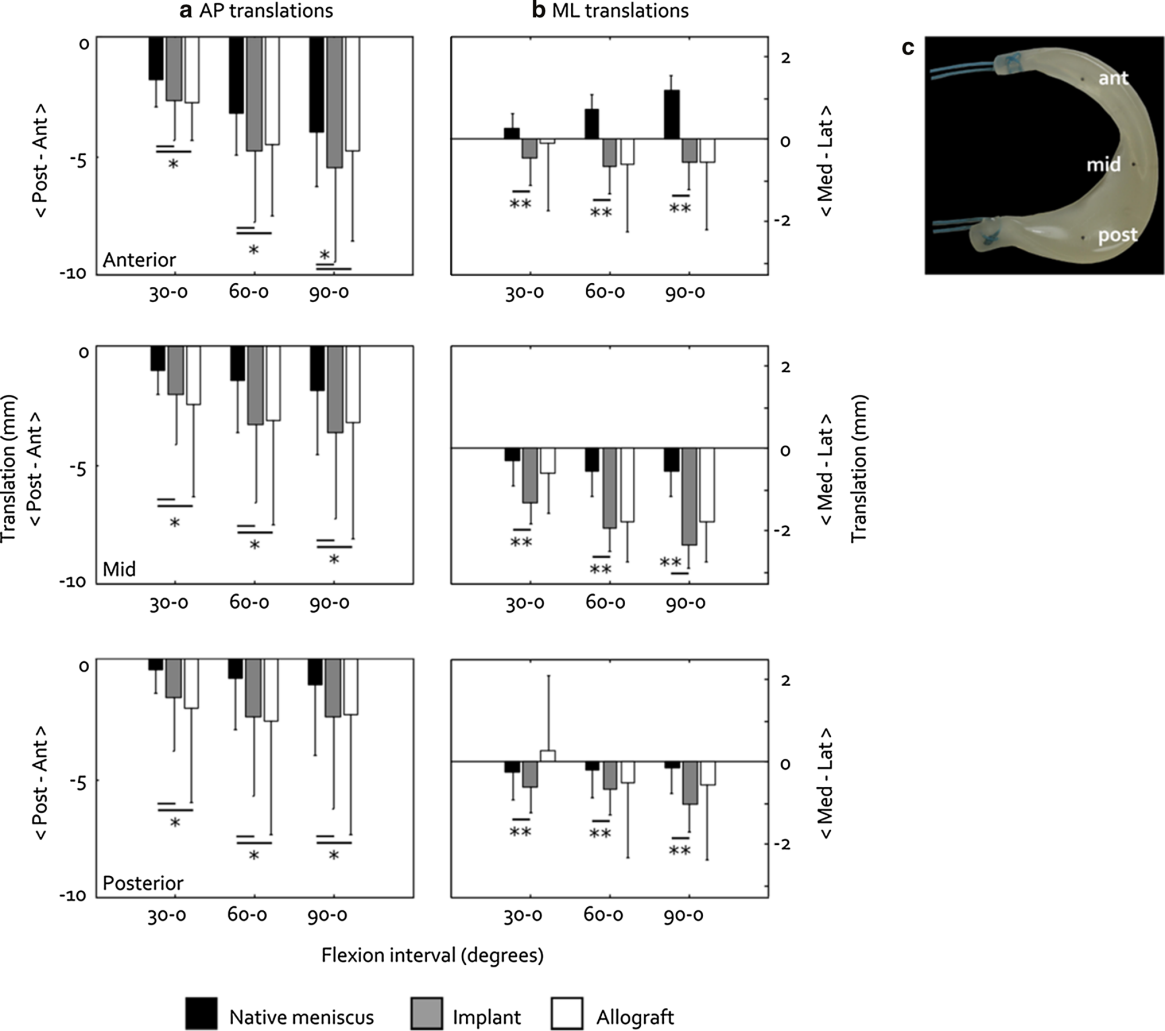
Meniscal translations (mean ± standard deviation) on the tibia plateau along **a** the AP and **b** the ML axis, for the native medial meniscus, implant and allograft. *p < 0.05 and **p < 0.001. **c** Location of the RSA markers in the anterior, mid and posterior regions of the implant, for which the displacements are displayed in (**a**) and (**b**)

For all three meniscal conditions, the translation patterns along the AP axis were similar; translation occurred in posterior direction and increased with flexion angle, and the anterior horn was more mobile than the mid-region (*p* = 0.002) and posterior horn (*p* < 0.001) (Fig. 3a). A significant increase in the posterior translation was observed when replacing the native meniscus with the implant (*p* < 0.001) or an allograft (*p* < 0.001). When comparing the outcomes for each location–flexion interval combination, the differences in posterior translation between the implant and allograft were never statistically significant, whereas those between the meniscal replacements and the native meniscus were always significant (Fig. 3a).

Translations along the ML axis were less regular, particularly for the anterior horn. With increasing flexion angle, the implant and allograft anterior horn both moved medially, whereas the native meniscus moved laterally (Fig. 3b). Also along the ML axis, meniscal mobility increased when the native meniscus was replaced by the implant (*p* < 0.001) or an allograft (*p* = 0.001). ML displacement of the implant and allograft was not significantly different. When comparing the outcomes for each location–flexion interval combination, the differences in ML translation between the implant and allograft were always non-significant, whereas the differences between the implant and native meniscus were always significant (Fig. 3b).

### Knee stability

**Fig. 4:**
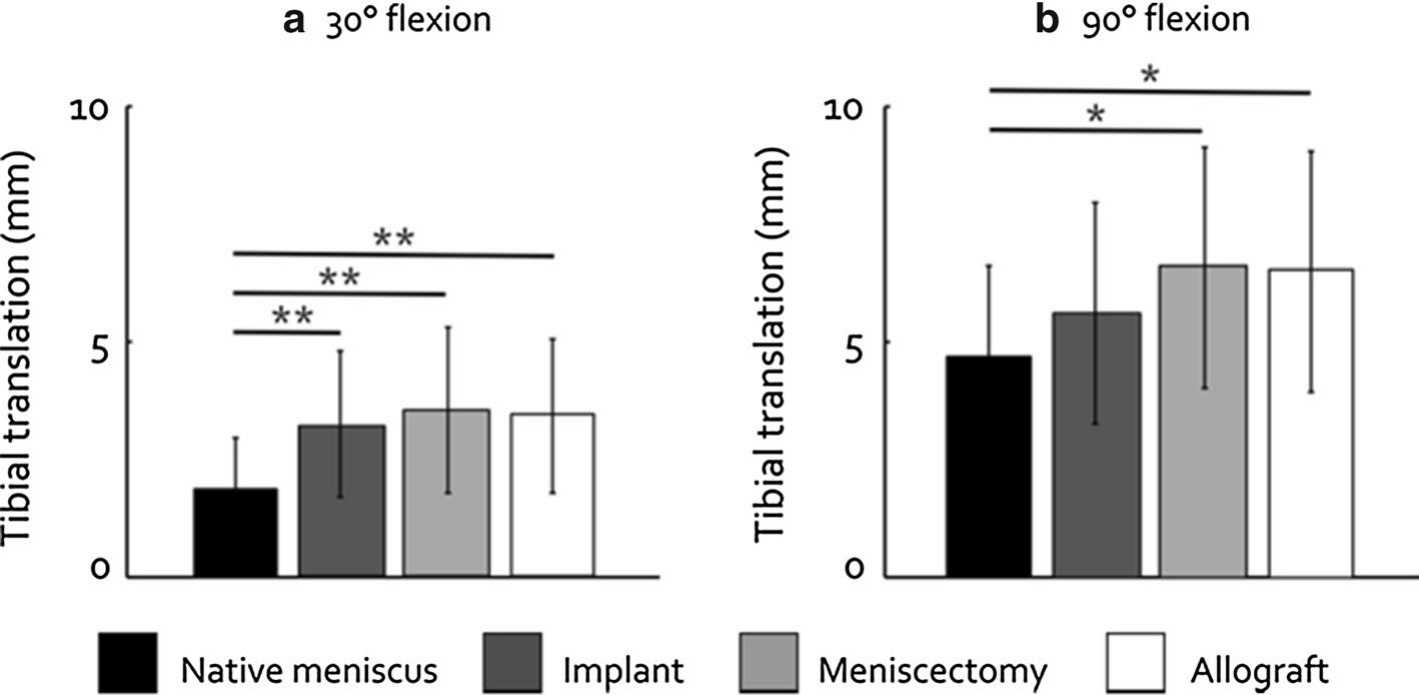
Knee AP laxity (mean ± standard deviation) in 30° flexion and 90° flexion. *p < 0.05 and **p < 0.001

**Fig. 5:**
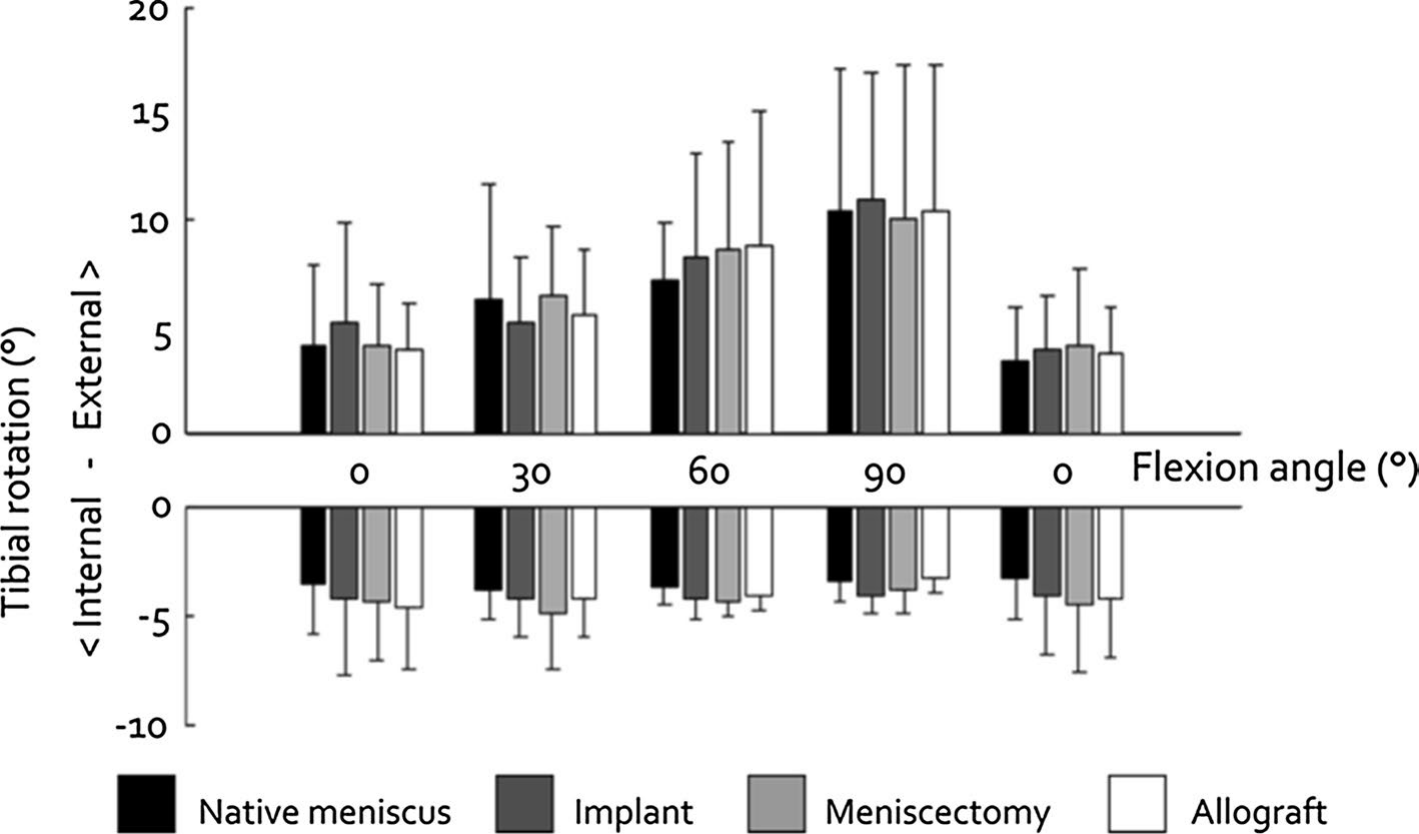
Tibial rotation (mean ± standard deviation) under influence of an externally (*top graph*) and internally (*bottom graph*) applied torque

The effect of changing the meniscal condition on AP laxity was larger in 30° flexion than in 90° flexion (Fig. 4). In 30° flexion, AP laxity was significantly increased for the implant, meniscectomy and allograft conditions (all *p* < 0.001). However, replacing the native meniscus by the implant did not significantly increase AP laxity in 90° flexion, whereas the increases in AP laxity found after meniscectomy and allograft transplantation were significant (*p* = 0.012 and *p* = 0.016). The changes in internal and external rotational laxity observed between the four meniscal conditions at each separate flexion angle were minor. The differences in rotational laxity between the first and second evaluation in 0° flexion for each meniscal condition were small and not significant (Fig. 5).

### Contact mechanics

**Fig. 6:**
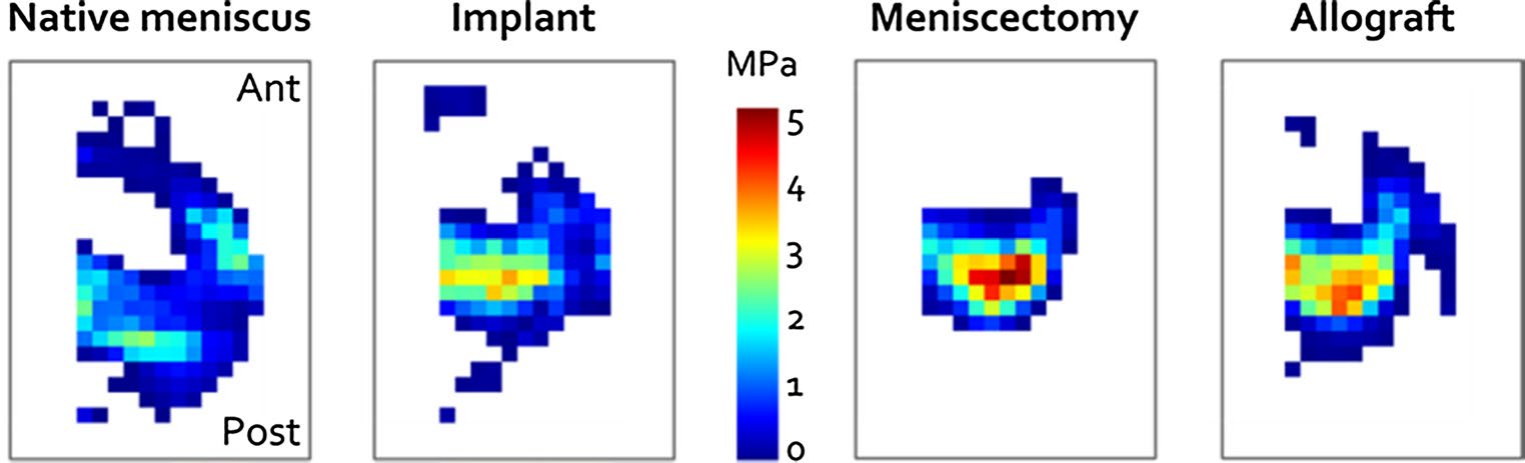
Example of the pressure distribution for the four different meniscal conditions

**Fig. 7:**
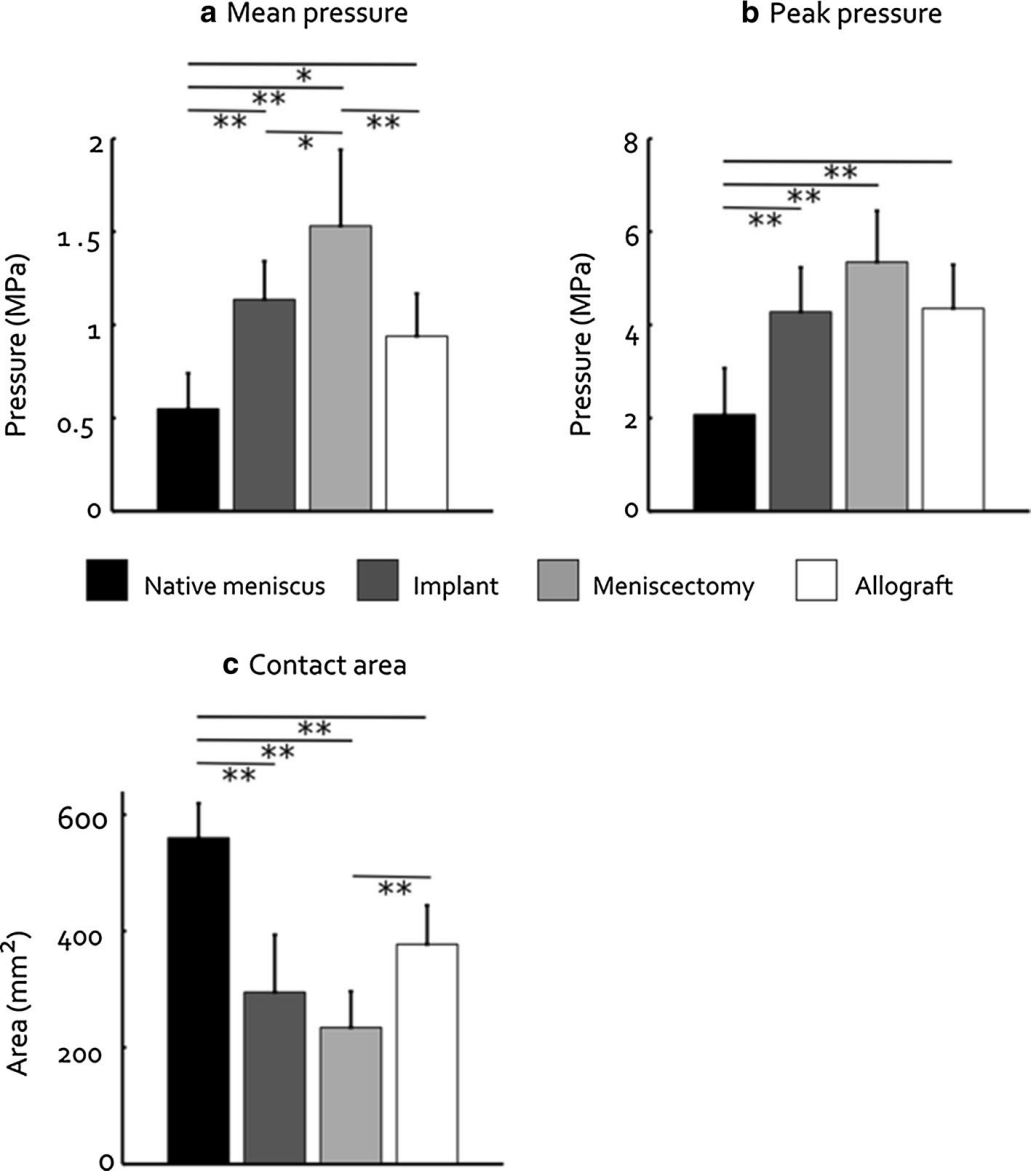
Meniscotibial contact mechanics (mean ± standard deviation) split into mean pressure, peak pressure and contact area. *p < 0.05 and **p < 0.001

A representative example of the pressure distribution on the tibia articular cartilage for the four meniscal conditions is shown in Fig. 6. The mean pressure for the native meniscus condition was significantly increased for the implanted knee (*p* < 0.001), after meniscectomy (*p* < 0.001) and after allograft transplantation (*p* = 0.019, Fig. 7). Both the implant condition and allograft condition did significantly reduce the mean contact pressure compared to the meniscectomy condition (*p* = 0.018 and *p* < 0.001, Fig. 7). Also the peak pressure was significantly elevated for the implant, meniscectomy and allograft conditions with respect to the native meniscus (all *p* < 0.001). The peak pressures for the implant and allograft were highly similar. The contact area on the tibia articular cartilage was largest for the native meniscus and was significantly reduced for the implant, allograft and meniscectomy conditions (all *p* < 0.001). Whereas the implant only slightly increased the contact area with respect to the meniscectomized condition, meniscal allograft transplantation resulted in a significant increase (*p* < 0.001).

## Discussion

The most important finding of the present study was that our anatomically shaped PCU total meniscus implant showed a similar biomechanical performance as a meniscal allograft, but was unable to restore the functionality of the native meniscus. The implant was more mobile than the native meniscus and not capable of restoring native contact mechanics. The implant significantly reduced the mean contact pressure relative to the meniscectomized knee, but was not capable of improving peak pressure, contact area or AP stability. On the other hand, the differences between the implant and allograft were small and non-significant, indicating that the implant could match the biomechanical performance of a meniscal allograft. Previously, it has been shown that meniscal allografts cannot fully restore the biomechanical functionality of the native meniscus [4, 20, 33]. Nevertheless, allografts have shown to be an effective treatment to relieve pain and resume activity for symptomatic meniscectomy patients [11, 18, 23]. This illustrates that it may not be necessary that a meniscal replacement fully restores the biomechanical outcomes to native meniscus levels.

The translations of the implant and allograft were always larger than those of the native meniscus, which may be due to the suture-only fixation technique. The sutures were knotted together to fix the meniscal replacements; however, the threads had the freedom to move within the tibial bone tunnels when the knee was loaded. Whereas the allograft had an additional peripheral fixation to the joint capsule, we did not observe significant differences in mobility between the allograft and implant. This corresponds to a previous observation that the integration between the medial meniscus and the MCL-joint capsule complex does not influence meniscal mobility [31].

The results on AP knee laxity of this study are in agreement with previous findings indicating that complete removal of the medial meniscus significantly increases anterior knee laxity, which suggests that the medial meniscus plays a primary role in AP knee stability [5, 27]. Although the maximum increase in anterior knee laxity was 89 % (meniscectomy versus native meniscus in 30° flexion), the corresponding absolute increase in anterior displacement was only 1.8 mm. In the scope of the classification by Daniel et al. [9] who considered a left to right AP laxity difference of smaller than 2 mm as normal, our changes in knee laxity are marginal.

Whereas the implant and allograft significantly decreased the mean pressure with respect to the meniscectomy condition, the peak pressure and contact area were not improved. These observations correspond with previously reported findings [4, 33]. The mean differences between the implant and native meniscus are close to the values reported by Alhalki et al. [4]. Recently, Wang et al. [33] reported inferior contact mechanics for allografts fixed with the suture-only technique compared to grafts fixed with bone plugs, which was associated with increased extrusion in the former condition. This suggests that our choice for the suture-only fixation technique may have been responsible for the deficient contact mechanics of the meniscus replacements. Clinical evaluations of allografts with suture-only and bone plug fixation have shown similar Lysholm, Tegner and VAS scores [1, 2]. However, contrary to meniscal allografts that over time will be reinforced by tissue ingrowth [18, 30], our implant will permanently depend on its initial fixation. This imposes extra demands on the fixation of the implant and suggests that future implant designs could benefit from a more rigid fixation strategy.

Some limitations should be addressed when interpreting the results of this study. Firstly, all tests were performed at room temperature, while PCU is known to be softer at body temperature and in a fully humidified environment [13]. To minimize the influence of temperature differences, the implant was kept in a 37 °C saline solution up to the moment of implantation. Secondly, the repeated measures design chosen for this study may have biased our results. However, similar values were recorded for tibial rotation at the beginning and end of a loading cycle. Therefore, we are confident that the outcomes of this study were not influenced by the repeated measures design. Lastly, the compressive loads used in this study were lower than physiological loads. Based on previous evaluations of meniscal motion and tibial contact mechanics at multiple load levels, it is expected that the absolute translations and pressures would be increased when higher loads would have been applied [24, 29]. However, it is not anticipated that an increased load would affect the relative differences between the four conditions in this study.

## Conclusions

The biomechanical performance of the novel PCU meniscal implant matched that of an allograft meniscus. Nevertheless, neither of the meniscal replacements could reproduce native meniscus performance or improve all aspects of contact mechanics compared to total meniscectomy. Based on the similarity between implant and allograft performance in this study, this implant may become an alternative to meniscal allograft transplantation.
